# Anti-Inflammatory and Neuroprotective Effects of Co-UltraPEALut in a Mouse Model of Vascular Dementia

**DOI:** 10.3389/fneur.2017.00233

**Published:** 2017-06-06

**Authors:** Rosalba Siracusa, Daniela Impellizzeri, Marika Cordaro, Rosalia Crupi, Emanuela Esposito, Stefania Petrosino, Salvatore Cuzzocrea

**Affiliations:** ^1^Department of Chemical, Biological, Pharmaceutical and Environmental Science, University of Messina, Messina, Italy; ^2^Endocannabinoid Research Group, Istituto di Chimica Biomolecolare, Consiglio Nazionale delle Ricerche, Pozzuoli, Naples, Italy; ^3^Epitech Group S.p.A., Saccolongo, Italy; ^4^Department of Pharmacological and Physiological Science, Saint Louis University School of Medicine, Saint Louis, CA, United States

**Keywords:** neuroprotection, palmitoylethanolamine, luteolin, oxidative stress, inflammation

## Abstract

Vascular dementia (VaD), the second most common cause of cognitive impairment in the population, is a disease that results from reduction in regional cerebral blood flow and involves oxidative stress and inflammation. Co-ultramicronized PEALut (co-ultra PEALut) is a new compound with beneficial effects, which include anti-inflammatory and antioxidant properties. Recently, co-ultraPEALut has been shown to exhibit neuroprotective effects in models of Parkinson’s disease, cerebral ischemia and Alzheimer’s disease. However, its effects on VaD remain unknown. Therefore, the purpose of the present study was to highlight the potential neuroprotective actions of co-ultraPEALut containing *N*-palmitoylethanolamine (PEA) and the antioxidant flavonoid luteolin (Lut) (10:1 by mass) in a mouse model of VaD induced by bilateral carotid arteries occlusion. At 24 h after VaD induction, mice were orally treated with 1 mg/kg co-ultraPEALut daily for 15 days. On the 15th day, brain tissues were processed for histological, immunohistochemical, Western blot, and immunofluorescent analysis. Our results clearly demonstrate that co-ultraPEALut improved learning, memory ability, locomotor activity, and the reciprocal social interaction. In addition, the mice subjected to VaD and treated with the co-ultraPEALut showed a reorganization of CA1 and CA3 regions of the hippocampus and restored the number of hippocampal neurons as evidenced by NeuN expression, a specific marker of neurons. Furthermore following carotid arteries ligation, mice treated with co-ultraPEALut showed a modification of proinflammatory, proapoptotic proteins and of oxidative stress as evidenced by the expression of IκB-α, NF-κB p65, Bax, Bcl-2, inducible nitric oxide synthase, and cyclooxygenase-2. In order, co-ultraPEALut treatment restored VaD-induced loss of brain-derived neurotrophic factor and neurotrophins 3 (NT-3) expression in mice. These results confirmed that the neuroprotective effects of co-ultraPEALut were associated with its anti-inflammatory and antioxidant properties.

## Introduction

An estimated 36 million people worldwide were afflicted with dementia. Among the subtypes of dementia, vascular dementia (VaD) is accounting for 15 to 20% of all cases of dementia (1). It results from a variety of causes, including cerebrovascular dysfunction. The common risk factors for VaD are diabetes, obesity, insulin resistance, hypertension, hyperhomocystinemia, and hyperlipidemia (2, 3). VaD happens when vessels that furnish blood to the brain become blocked or restricted and consequently the supply of blood carrying oxygen to the brain is abruptly cut off. However, not all people with stroke will develop VaD. Avoiding and controlling risk factors such as diabetes, high blood pressure, smoking, and high cholesterol can help curb the risk of VaD (4, 5). Many cases of VaD start with slight early warning signs: slowness of though, difficulty with planning trouble with attention, and concentration mood or behavioral changes. Left untreated, the symptoms may continue to get worse (6). Currently, no available treatments can repair the damage of VaD once it happened (7). The Food and Drug Administration (FDA) has not approved any drugs specifically to treat changes in judgment, planning, memory and other thought processes caused by VaD. However, certain medications approved by the FDA to treat these symptoms in Alzheimer’s disease may also help people with VaD to the same modest extent they help those with Alzheimer’s (8). After blocking of the carotid arteries, a significant reduction in regional cerebral blood flow causes deprivation of oxygen and glucose. These events lead to the activation of neuroinflammation, oxidative, and nitrosative stress that are the main causes of VaD (9, 10). A constitutively low concentration of oxidants is necessary and they behave as signaling molecules for various functions such as regulation of vascular tone, monitoring of oxygen tension, and erythropoietin production (11, 12). A lot of oxygen free radicals and their derivatives are generated after stroke, including superoxide anions $\left({\text{O}}_{2}^{•-}\right)$, hydrogen peroxide (H_2_O_2_), and hydroxyl radicals (^⋅^OH). ${\text{O}}_{2}^{•-}$ can react with nitric oxide (NO) to produce peroxynitrite (ONOO^−^) (NO + ${\text{O}}_{2}^{•-}$ → ONOO^−^), which is a strong oxidative radical that causes protein nitration and dysfunction (13). NO generated from neuronal nitric oxide synthase nitrosylates protein, which leads to cell dysfunction (14). Therefore, the purpose of this study was to evaluate the neuroprotective properties of a co-ultramicronized compound constituted by the association of *N*-palmitoylethanolamine (PEA), an endogenous fatty acid amide members of N-acylethanolamines family, with the vegetable flavonoid luteolin (Lut) called co-ultramicronized PEALut (co-ultra PEALut), in a mouse model of VaD at 15 days after the carotid arteries occlusion. PEA is plentiful in the central nervous system (CNS) and is produced by glial cells (15–17). PEA anti-inflammatory and neuroprotective effects have been studied mainly in models of peripheral neuropathies (18). In addition, previous studies have showed that PEA treatment significantly decreased the inflammation in mouse experimental models of spinal cord injury (19) and traumatic brain injury (20), as well as in CNS pathologies like PD where neuroinflammation plays a key role (21). However, PEA lacks direct antioxidant capacity to avoid oxidative stress and counteract damage to DNA and proteins, all of which are significant events in CNS diseases. Lut belongs to the family of flavonoids, which are largely distributed in plants, and which play an important role in the defense against microorganisms, insects and ultraviolet radiation (22). Given their large quantity in foods, flavonoids have antioxidant effect and antimicrobial function (23). Lut exhibits excellent antioxidant, cytoprotective, and pharmacological properties, by suppressing the production of tumor necrosis factor alpha, interleukins, free radicals (reactive oxygen and nitrogen species), and their signal transduction pathways (24–26). Moreover, in *in vivo* studies Lut diminishes increased vascular permeability and is efficient in animal models of CNS inflammation (27). Interestingly, while PEA has not any antioxidant effects *per se*, its coultramicronization with the flavonoid Lut is more efficient than either molecule alone. Previously, the association of these two molecules, in a fixed ratio of 10:1 in mass, has revealed a strong neuroprotective activity (28). Recently, co-ultraPEALut has shown an important role in the neurodegenerative diseases (Alzheimer’s and Parkinson’s) (29, 30) and in traumatic CNS injury (brain and spinal cord injury) (31, 32). In all these cases, this compound was able to reduce significantly the neurodegeneration and neuroinflammation that characterized these pathologies. About this, we hypothesized that co-ultraPEALut can play an important role on improving dementia following carotid arteries occlusion.

## Materials and Methods

### Animals

CD1 mice (male 25–30 g; Envigo, Milan, Italy) and Sprague Dawley rats (male 200–250 g; Envigo, Milan, Italy) were accommodated in a controlled environment and equipped with regular rodent food and water. Mice and rats were accommodated in steel cages in a room kept at 22 ± 1°C with a 12-h light, 12-h dark cycle. Mice and rats were acclimatized to their habitat for 1 week and they had *ad libitum* access to water of faucet rand rodent standard diet. The University of Messina Review Board for the care of animals approved the research. All animal experiments observe the regulations in Italy (D.M. 116192) as well as the EU regulations (O.J. of E.C. L 358/1 12/18/1986).

### First Step of the Study: Pharmacokinetics of PEA

#### Healthy Rats

In order to measure the brain penetration of ultramicronized PEA, we carried out a simple pharmacokinetic study using ultramicronized-[^13^C]_4_-PEA (UM-PEA), whose quantification by our LC-MS method would not be biased by the presence of endogenous, unlabeled PEA. UM-[^13^C]_4_-PEA was orally administered at the dose of 30 mg/kg. Rats were sacrificed after 0 min from administration of vehicle [carboxymethylcellulose (CMC) 2.5% p/p in water], 5 and 15 min from administration of UM-PEA. Subsequently, brains were removed, subjected to extraction, purification, and quantification of [^13^C]_4_-PEA by liquid chromatography-atmospheric pressure chemical ionization-mass spectrometry (LC-APCI-MS) analysis.

#### [^13^C]_4_-PEA Measurement by LC-APCI-MS

Liquid chromatography-atmospheric pressure chemical ionization-mass spectrometry analysis of [^13^C]_4_-PEA levels was carried out as previously described (33–35). The only difference being that 10 pmol of *N*-heptadecanoyl-ethanolamine were added as internal standard instead of [^2^H]_4_-PEA. In fact, we could not use commercially available [^2^H]_4_-PEA for these measurements since it has the same MW as [^13^C]_4_-PEA. Therefore, we had to use a chemically distinct internal standard, the C17 homolog of PEA, *N*-heptadecanoyl-ethanolamine. Briefly, brains were homogenized in a solution of chloroform/methanol/Tris–HCl 50 mM pH 7.4 (2:1:1 by vol.) containing 10 pmol of *N*-heptadecanoyl-ethanolamine as internal standard. The lipid-containing organic phase was prepurified by open-bed chromatography on silica gel, and fractions obtained by eluting the column with a solution of chloroform/methanol (90:10 by vol.) were analyzed by LC-APCI-MS by using a Shimadzu HPLC apparatus (LC-10ADVP) coupled to a Shimadzu (LCMS-2020) quadrupole MS via a Shimadzu APCI interface. LC-APCI-MS analysis of [^13^C]_4_-PEA was carried out in the selected ion monitoring mode, using *m/z* values of 314 and 304 (molecular ions + 1 for the standard and [^13^C]_4_-PEA, respectively), and retention times were 17 and 13 min, respectively. [^13^C]_4_-PEA levels were calculated on the basis of their area ratio with the internal standard signal areas, and the amounts (pmol) were normalized per g of tissue.

### Second Step of the Study: Animal Model of VaD Induction

The mice were anesthetized by xylazine and ketamine [0.16 and 2.6 mg/kg body weight, respectively, given intraperitoneal (i.p.)] and then the anterior cervical skin was disinfected with 75% alcohol. A ventral midline skin cut was made in the neck zone after moderate tweezing of neck muscles and just above the sternal bone, carotid arteries were identified and then a wire was passed below each carotid artery for closure. The bilateral carotid arteries were locked by ligation for 10 min, and then released for 10 min, and this was repeated three times. The threading was then removed and the incision sutured. After surgery, mice were located under a heating lamp for the prevention of hypothermia until complete recovery from overall anesthesia. The mice were placed in cages for rearing. 15 days after surgery mice were sacrificed by decapitation. Brains were dissected out, sectioned, and processed (36).

#### Experimental Groups

The animals were arbitrarily divided into the following groups:

*Group 1*: Sham + vehicle = mice were subjected the same surgical procedure without carotid arteries ligation and treated orally with vehicle [CMC (1.5% wt/vol in saline)] for 15 days (*N* = 10).
*Group 2*: Sham + co-ultraPEALut = same as the Sham + vehicle group, but co-ultraPEALut [1 mg/kg orally, dissolved in CMC (1.5% wt/vol in saline)] was administered 24 h after the surgical procedure without carotid arteries ligation and continuing for 15 days after (data not shown) (*N* = 10).
*Group 3*: VaD + vehicle = the mice were subjected to the aforementioned modeling method and treated with vehicle as described for Sham + vehicle group (*N* = 10).
*Group 4*: VaD + co-ultraPEALut = same as the VaD + vehicle group, but co-ultraPEALut [at a dose of 1 mg/kg orally, dissolved in CMC (1.5% wt/vol in saline)] was administered 24 h after the surgical procedure and continuing for 15 days after (*N* = 10).

The dose and the animal model used were based on previous *in vivo* studies (36, 37).

#### Preparation of Nuclear and Cytosolic Extracts from Brain and Western Blot Analysis

To perform Western blot analysis, the mice were anesthetized by xylazine and ketamine (0.16 and 2.6 mg/kg body weight, respectively, given i.p.) and after decapitated with large bandage scissors. Brains of each mouse were quickly removed and suspended in extraction Buffer A comprising 20 mM leupeptin, 0.15 mM pepstatin A, 1 mM sodium orthovanadate, 0.2 mM PMSF, homogenized for 2 min, and centrifuged at 12,000 rpm at 4°C for 4 min. Supernatants represented the cytosolic portion. The pellets, which contains nuclei, were resuspended in Buffer B comprising 10 mM Tris–HCl pH 7.4, 150 mM NaCl, 1 mM EGTA, 1% Triton X-100, 1 mM EDTA, 0.2 mM PMSF, 20 mm leupeptin, and 0.2 mM sodium orthovanadate. After centrifugation for 10 min at 12,000 rpm at 4°C, the supernatants containing the nuclear protein. Protein concentrations were assessed by the Bio-Rad protein assay using bovine serum albumin as standard. The expression of inducible nitric oxide synthase (iNOS), Bax, Bcl-2, cyclooxygenase-2 (COX-2), and IκB-α were quantified in cytosolic fractions. NF-κBp65 was quantified in nuclear fractions collected 15 days after VaD-induction. The filters were probed with specific antibodies for rabbit polyclonal anti-IκB-α (1:500; Santa Cruz Biotechnology), rabbit polyclonal anti-NF-κB p65 (1:1,000; Santa Cruz Biotechnology), mouse monoclonal anti-iNOS (1:1,000; BD Trasduction), mouse monoclonal anti-COX-2 (1:1,000; Cayman), rabbit polyclonal anti-Bax (1:500; Santa Cruz Biotechnology), and rabbit polyclonal anti-Bcl-2 (1:500; Santa Cruz Biotechnology) were mixed in 1 × phosphate-buffered saline (PBS), 5% w/v non-fat dried milk, 0.1% Tween-20 and incubated at 4°C overnight. Membranes were then incubated with peroxidase-conjugated goat antirabbit IgG or peroxidase-conjugated bovine antimouse IgG secondary antibody (1:2,000; Jackson ImmunoResearch) for 1 h at room temperature. To make sure that blots were loaded with equal amounts of protein lysates, they were also incubated with the antibody agonist β-actin or laminin antibody (1:5,000; Santa Cruz Biotechnology). Signals were detected with enhanced chemiluminescence detection system reagent giving to the company’s instructions (SuperSignal West Pico Chemiluminescent Substrate, Pierce). Relative expression of protein bands was quantified by densitometry with BIORAD ChemiDoc™ XRS + software and standardized to β-actin levels. Images of blot signals (8 bit/600 dpi resolution) were transferred to analysis software (Image Quant TL, v2003).

#### Immunohistochemical Localization of Bax, Bcl-2, Poly-ADP-Ribose, and Nitrotyrosine

At the end of the experiment, brains were before fixed in 10% (w/v) PBS-buffered formalin and then 7 µm sections were prepared from paraffin-fixed tissues. After deparaffinization, endogenous peroxidase was reduced with 0.3% (v/v) H_2_O_2_ 60% (v/v) methanol for 30 min. The sections were permeabilized with 0.1% (v/v) Triton X-100 in PBS for 20 min. Non-specific adsorption was minimized incubating the section in 2% (v/v) normal horse serum in PBS for 20 min. Endogenous biotin or avidin binding points were blocked by sequential incubation with avidin and biotin for 15 min (Vector Laboratories, Burlingame, CA, USA). Slices were incubated overnight with anti-Bax antibody (Santa Cruz Biotechnology, 1:100 in PBS, v/v), anti-Bcl-2 antibody (Santa Cruz Biotechnology, 1:500 in PBS, v/v), antipoly ADP-ribose (anti-PAR) antibody (Santa Cruz Biotechnology, 1:100 in PBS, v/v), and antinitrotyrosine (Millipore, 1:500 in PBS, v/v). Sections were cleaned with PBS and incubated with secondary antibody. Specific category was identified with a biotin-conjugated goat antirabbit IgG and avidin-biotin peroxidase complex (Vector Labs Inc., Burlingame, CA, USA). To verify the binding specificity for different antibodies, some sections were also incubated with only primary or secondary antibody; no positive staining was observed in these sections. The sections were quantitatively evaluated for a variance in immunoreactivity by computer-assisted color image investigation (Leica QWin V3, Cambridge, UK). Immunoreactivity was quantized within five random fields at ×20 and ×40 magnifications. The fraction of positive staining as a function of total tissue area was determined (38). In Sham mice, the central zones of corresponding tissue portions were taken as reference points and an equivalent number of optical fields were calculated. All the immunocytochemistry analysis was carried out without knowledge of the treatments.

#### Immunofluorescence of NeuN, Brain-Derived Neurotrophic Factor, Neurotrophins 3, GFAP, and Iba-1

After deparaffinization and rehydration, detection of NeuN, brain-derived neurotrophic factor (BDNF), neurotrophins 3 (NT-3), GFAP, and Iba-1 was carried out after boiling for 1 min in 0.1 M citrate buffer. Non-specific adsorption was reduced by incubating the section for 20 min in 2% (vol/vol) normal goat serum in PBS. Sections were incubated with mouse monoclonal anti-NeuN (Millipore; 1:100 in PBS, v/v), with mouse monoclonal anti-GFAP (Santa Cruz Biotechnology; 1:200 in PBS, v/v), with mouse monoclonal anti-Iba-1 (Santa Cruz Biotechnology; 1:200 in PBS, v/v), with rabbit polyclonal anti-BDNF (Santa Cruz Biotechnology; 1:200 in PBS, v/v), or with rabbit polyclonal anti-NT-3 (Santa Cruz Biotechnology; 1:100 in PBS, v/v) antibodies in a humidified chamber O/N at 37°C. Sections were cleaned with PBS and were incubated with secondary antibody TEXAS RED-conjugated antirabbit Alexa Fluor-594 antibody (1:1,000 in PBS, v/v Molecular Probes, UK) and with FITC-conjugated antimouse Alexa Fluor-488 antibody (1:2,000 v/v Molecular Probes, UK) for 1 h at 37°C. Sections were laved and for nuclear staining 4′,6′-diamidino-2-phenylindole (Hoechst, Frankfurt; Germany) 2 µg/mL in PBS was added. Sections were seen and photographed by a Leica DM2000 microscope (Leica, Milan Italy). Optical portions of fluorescence samples were acquired using an Ar laser (458 nm) and a HeNe laser (543 nm), a laser UV (361–365 nm) at a 1 min, 2 s scanning speed with up to eight averages; 1.5-µm sections were acquired using a pinhole of 250. Examining the most intensely labeled pixels and applying backgrounds that allowed clear image of structural details while keeping the highest pixel intensities close to 200 established contrast and illumination. The same backgrounds were used for all images acquired from the other samples that had been managed in parallel. Digital images were collected and figure montages arranged using Adobe Photoshop CS6 (Adobe Systems, Milan, Italy).

#### Behavioral Testing

Behavioral evaluations on all mice were made 1 day prior to, and 15 days after, surgical procedure with carotid arteries ligation.

##### Social Interaction Test

Qualitative and quantitative impairments in social interaction are important clinical features of dementia (39). For this reason, behavioral neuroscientists use standardized scoring methods to quantitate various types of social interactions in mice. Preference for social approach was measured using the three-chambered apparatus (40). The apparatus was a rectangular box made of polycarbonate (80 cm × 31.5 cm) and was divided into three compartments by partitions with openings, which allowed free access to all compartments. The paradigm consisted of three trials each lasting for 10 min. Initially, mice were habituated to the empty arena for 5 min after which empty wire cages were introduced and the mice were allowed to inspect these cages for another 10 min. The second phase of the test measured social preference involving a 10-min session where the experimental mouse was exposed to an inanimate object, one of the empty wired cages and a wired cage covering a stimulus mouse. The placement of both social and non-social targets was counterbalanced between experimental subjects. Time spent engaging in investigatory behavior with the novel mouse and frequency of investigatory behavior with the novel mouse was monitored by a color charge-coupled device camera (Sony DXC-151A) in order to observe social approach and preference. Parameters measured were the number of active contacts initiated by the target animal, mean duration per contact, total duration of contact, and total distance traveled were measured. The number of active contacts was defined as follows. Images were acquired at one frame per second, and the distance moved between two consecutive frames was estimated for each mouse. If the two mice in mutual contact and the distance traveled by either mouse was longer than 5 cm, the behavior was considered to be “active contact.” The mouse that traveled a longer distance from the previous frame was considered to have approached the other subject actively. All testing happened during the dark phase (21:00–03:00 h) under illumination with red light.

##### Novel Object Recognition Test

Mice were tested in a round open field (OF, 40 cm diameter) situated in a room with dim lighting. Briefly, mice were familiarized to the OF in the absence of the objects for 10 min/day over 2 days. During the training period, mice were placed in the OF with two identical objects for 10 min. The retention test was performed 90 min (short-term memory) and 24 h (long-term memory) after training by placing the mice back to the OF for a 5 min session and by randomly exchanging one of the familiar objects with a novel one. Each session was videotaped from above and the dependent measure included time spent investigating an object. Mice were considered to be exploring an object when it was actively sniffing the object or if the nose was investigating the familiar object in the test period, which implies that they recognized the object before presented (41).

##### Open Field

Locomotor activity was observed in an OF for 5 min, a Plexiglas box 50 cm × 50 cm with its floor separated into 16 squares. Four squares were defined as the center and 12 squares along the walls as the periphery. Each mouse was gently located in the center of the box and movement was scored as a line crossing when a mouse removed all paws from one square and entered another. Before each test, the chamber was washed with water containing a detergent. The animals’ behavior was recorded. The line crossings and the period spent in the center were calculated and scored.

All the behavioral studies were performed in a blinded fashion.

#### Light Microscopy

For histopathological examination, standard hematoxylin and eosin (H&E) staining was performed. Briefly, 15 days after the surgery, mice in each group were selected and were decapitated after anesthesia. The brain was quickly removed and was fixed in 4% formalin. Following dehydration with ethanol and embedding with paraffin, 7 µm sections were made, followed by H&E staining. The hippocampal CA1 and CA3 regions were then observed under an optical microscope (Leica QWin V3, Cambridge, UK). Ischemic neuronal damage was graded on a scale of 0 = normal, 1 = a few (<30%) neurons damaged, 2 = many neurons (30 to 70%) damaged, and 3 = majority of neurons (>70%) damaged (42). All the histological studies were performed in a blinded fashion.

### Materials

Unless otherwise indicated, all compounds were obtained from Sigma-Aldrich, while co-ultraPEALut and [^13^C]_4_-PEA were a kind gift from Epitech Group Spa (Saccolongo, Italy). All other substances were of the highest commercial grade available. All stock solutions were prepared in non-pyrogenic saline (0.9% NaCl, Baxter, Milan, Italy) or 10% dimethyl sulfoxide.

### Statistical Evaluation

All values in the figures and the text are expressed as mean ± SEM. Results shown in the figures are representative of at least three experiments performed on different *in vivo* experimental days. In each experiment, we used five animals per group, unless otherwise indicated. The results were analyzed by one-way analysis of variance followed by a Bonferroni *post hoc* test for multiple comparisons. A *p*-value of less than 0.05 was considered significant.

## Results

### Levels of [^13^C]_4_-PEA in the Brain of Healthy Rats

**Figure 1:**
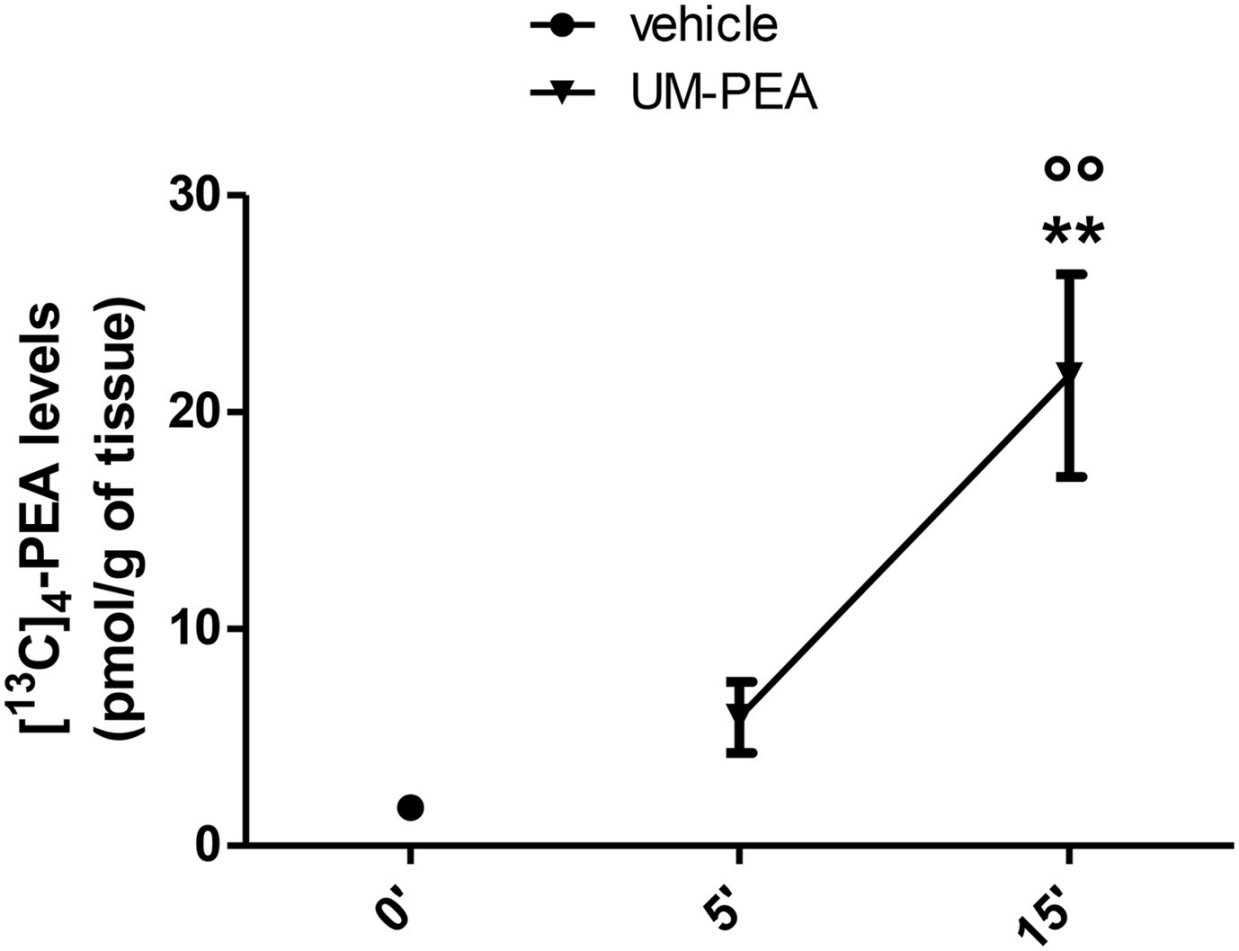
**Brain [^13^C]_4_-*N-palmitoylethanolamine* (PEA) levels in healthy rats**. Levels of [^13^C]_4_-PEA in the brain of healthy rats after oral administration of 30 mg/kg of ultramicronized-[^13^C]_4_-PEA (UM-PEA). Data are expressed as mean ± SEM of five animals for each group and analyzed by one-way ANOVA followed by Bonferroni’s multiple comparison test. **p < 0.01 for 0′ versus 15′; °°*p* < 0.01 for 5′ versus 15′.

**Figure 2:**
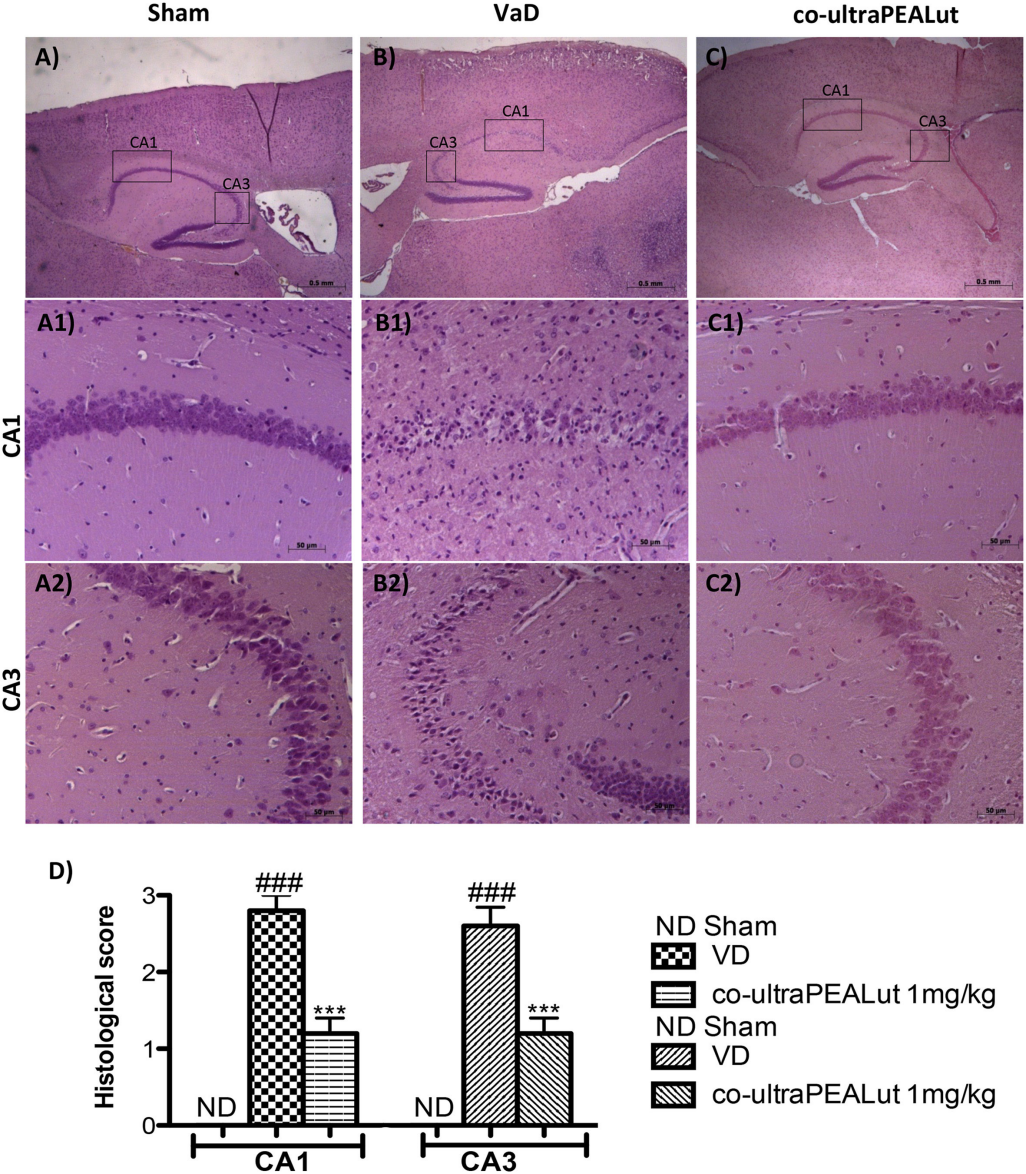
**Effects of co-ultraPEALut on histological parameters in hippocampal area after carotid arteries occlusion**. Control mice showed large numbers of nerve cells with closely arranged, neat and rounded nuclei in hippocampal **(A)**, in particular in CA1 and CA3 regions **(A1,A2)**, whereas vascular dementia (VaD) mice showed low number of nerve cells and hippocampal disorganized structure **(B,B1,B2)**. Co-ultraPEALut treatment restored the number of neurons to a level comparable to control mice **(C)** and mice showed a reorganization of hippocampal CA1 and CA3 regions **(C1,C2)**. These data are also visible in the histological score **(D)**. ***p < 0.001 vs. Sham; ^###^*p* < 0.001 vs. VaD, as determined by one-way ANOVA followed by Bonferroni *post hoc* test.

Our results, shown in Figure 1, indicate that, after oral administration of 30 mg/kg of UM-[^13^C]_4_-PEA, the peak concentration of [^13^C]_4_-PEA was found at 15 min in the brain of healthy rats (21.68 ± 4.67 pmol/g) (Figure 1). In fact, we observed that, at this time point, [^13^C]_4_-PEA levels were (1) 12-fold higher than [^13^C]_4_-PEA levels found at time 0 (1.77 ± 0.49 pmol/g) (Figure 1) and (2) 4-fold higher than [^13^C]_4_-PEA levels found after 5 min from administration of UM-[^13^C]_4_-PEA (5.93 ± 1.63 pmol/g) (Figure 1). These finding indicates that oral treatment with 30 mg/kg of UM-PEA can result in low-medium nanomolar brain concentrations of PEA already shortly after administration.

### Effect of Co-ultraPEALut on Histological Parameters

To evaluate in the mice the severity of the damage at the level of the hippocampal area 15 days after injury, the sections obtained from each group were stained with H&E. Sections from normal control mice showed a normal architecture in hippocampal. Nerve cells were numerous, closely organized and had rounded nuclei (Figure 2A, for hippocampal CA1 and CA3 regions see magnification higher A1, A2, and relative histological analysis Figure 2D). Reduced number of nerve cells, disorganized, heavily stained were found in the hippocampal of all mice at 15 days after the injury (Figure 2B, for hippocampal CA1 and CA3 regions see magnification higher B1, B2, and relative histological analysis Figure 2D). In contrast, brain sections from mice treated with co-ultraPEALut showed a marked reorganization of hippocampal CA1 and CA3 regions; treatment also restored the number of hippocampal neurons (Figure 2C, for hippocampal CA1 and CA3 regions see magnification higher C1, C2, and relative histological analysis Figure 2D ***p < 0.001 vs. Sham; ^###^*p* < 0.001 vs. VaD).

### Behavioral Measurements Following Carotid Arteries Ligation

#### Reciprocal Social interactions

**Figure 3:**
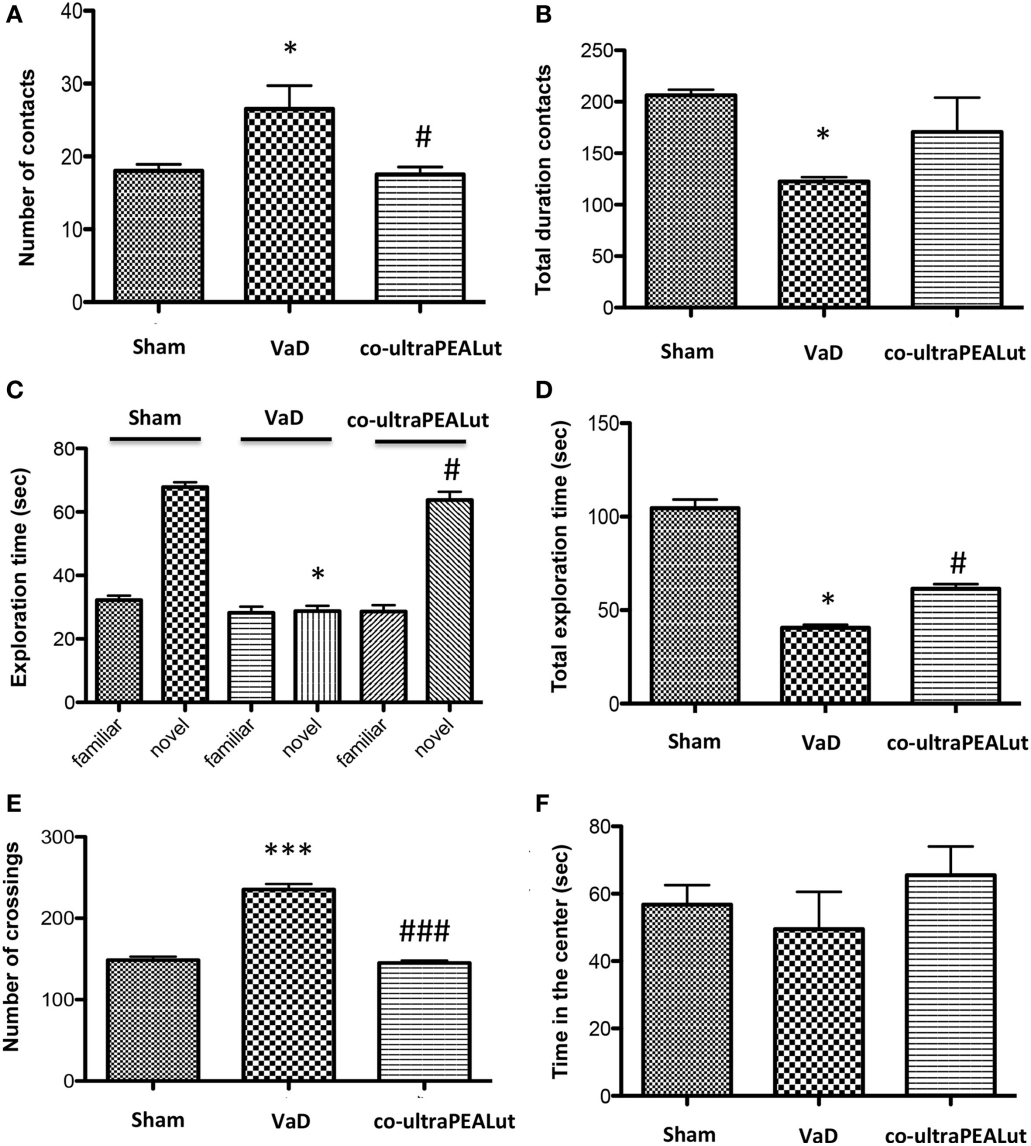
**Effect of co-ultraPEALut on behavioral changes in vascular dementia (VaD) mice**. **(A,B)** Effects of co-ultraPEALut treatment on Reciprocal Social Interactions. **(A)** The number of contacts was significantly increased in VaD mice; while number of social contacts was decreased in mice treated with co-ultraPEALut. *p < 0.05 vs. Sham; ^#^*p* < 0.05 vs. VaD. **(B)** The total duration contacts were significantly decreased in VaD mice, while they increased in mice treated with co-ultraPEALut. *p < 0.05 vs. Sham. **(C,D)** Effects of co-ultraPEALut treatment on memory deficit using the Novel Object Recognition. Results are calculated by the time expended exploring novel object at each intertrial interval and the time spent investigating the familiar object at each intertrial interval. **(C)** In the first time, controls and VaD animals showed no significant difference in exploration time of objects; while mice spent more time exploring the novel object after co-ultraPEALut treatment. *p < 0.05 vs. Sham; ^#^*p* < 0.05 vs. VaD. **(D)** VaD animals showed significantly reduced of memory for the objects; while in mice treated with co-ultraPEALut the total exploration time of objects returned to normal values. *p < 0.05 vs. Sham; ^#^*p* < 0.05 vs. VaD. **(E,F)** Effects of co-ultraPEALut treatment on locomotor behaviors in open field. Locomotor deficit is calculated as mean of the total time in the center in seconds. **(E)** The number of crossing was increased in VaD mice, while this number was significantly reduced in mice treated with co-ultraPEALut. ***p < 0.001 vs. Sham; ^###^*p* < 0.001 vs. VaD. **(F)** Regarding, carotid arteries occlusion decreased the time spent in the center of arena. This alteration was reversed by administration of co-ultraPEALut. Data were determinated by one-way ANOVA followed by Bonferroni *post hoc* test.

Social behavior represents a common deficit in VaD. Following the introduction of the unfamiliar mouse into the three-chamber test arena, we observed that the number of contacts was significantly increased in mice of VaD group when compared to control; while number of social contacts was decreased in mice treated with co-ultraPEALut (Figure 3A; *p < 0.05 vs. Sham; ^#^*p* < 0.05 vs. VaD). On the contrary the total duration contacts with unfamiliar mouse were significantly decreased in VaD mice, while they increased in mice treated with co-ultraPEALut (Figure 3B; *p < 0.05 vs. Sham).

#### Novel Object Recognition

Using the Novel Object Recognition test we evaluated changes in cognitive function. Mice were tested at 15 days after the injury and compared to Sham-operated controls. During training, controls and VaD animals showed no significant difference in exploration time of objects; while mice spent more time to discover the novel object in the test session after co-ultraPEALut treatment (Figure 3C; *p < 0.05 vs. Sham; ^#^*p* < 0.05 vs. VaD). At 15 days following carotid arteries ligation, VaD animals showed significantly reduced preference for the novel object, indicating compromise of cognitive function; while in mice treated with co-ultraPEALut the function within the novel object recognition test returned to normal values (Figure 3D; *p < 0.05 vs. Sham; ^#^*p* < 0.05 vs. VaD).

#### Open Field

To measure locomotor activity we used an OF test. After 15 days from carotid arteries ligation, number of crossing was increased in VaD animals, while crossings were significantly reduced in mice treated with co-ultraPEALut (Figure 3E; ***p < 0.001 vs. Sham; ^###^*p* < 0.001 vs. VaD). In contrast, the occlusion of the carotid arteries has led to significantly decrease the time spent in the center of arena in mice of VaD group. Such alteration was then reversed by co-ultraPEALut treatment (Figure 3F).

### Co-ultraPEALut Reduces the Activation of NF-κB Following Carotid Arteries Ligation

**Figure 4:**
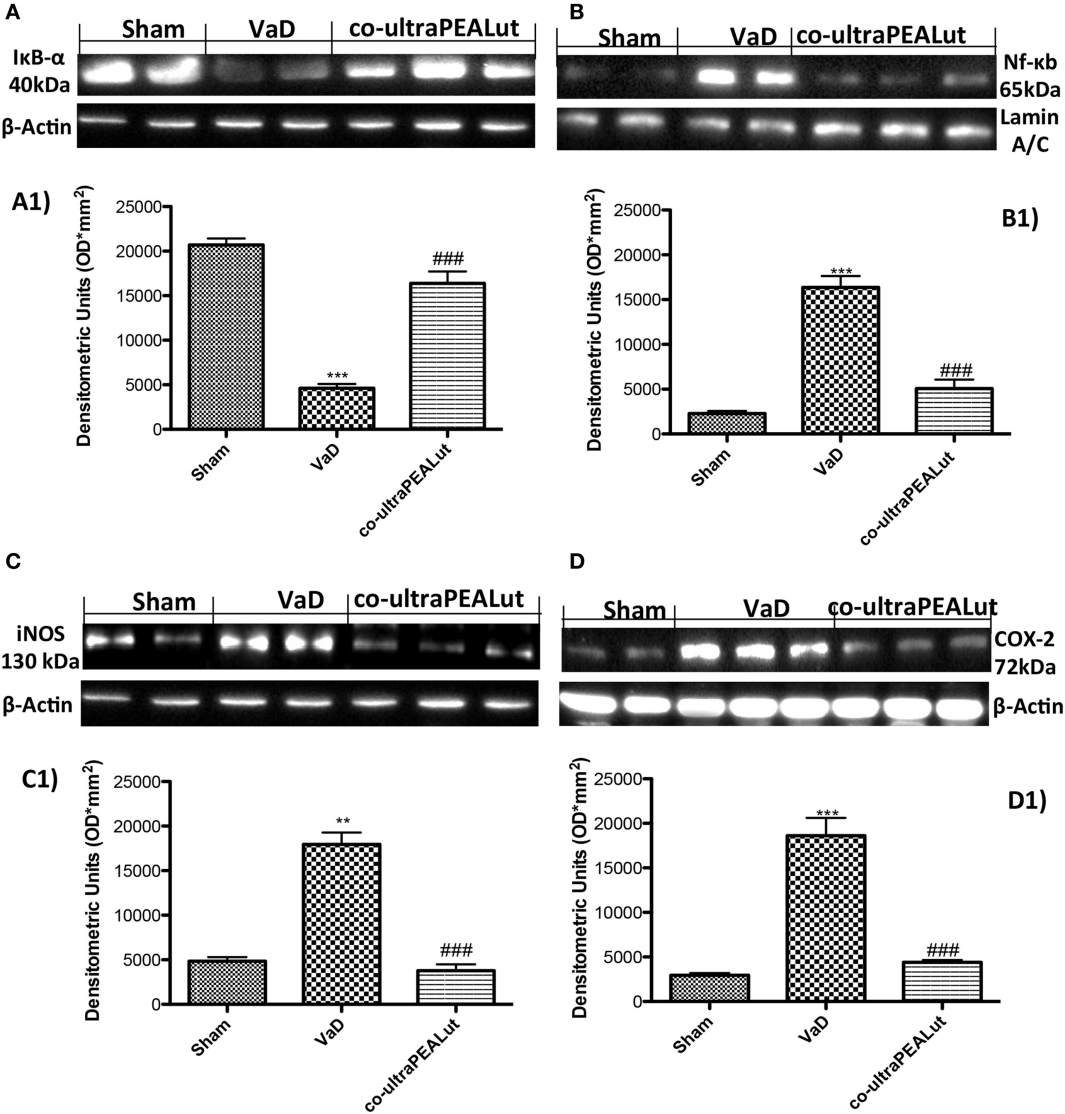
**Effects of co-ultraPEALut treatment on NF-κB pathways, inducible nitric oxide synthase (iNOS) and cyclooxygenase**-**2 (COX-2) expression**. Western blot analysis for IκB-α and NF-κB proteins in the brain were detected at 15 days after carotid arteries occlusion. IκB-α levels were reduced substantially in mice treated with co-ultraPEALut compared to VaD mice **(A,A1)**. NF-κB p65 levels in the VaD mice were increased significantly compared to Sham mice **(B)**. Low levels of NF-κB p65 were found in co-ultraPEALut treated animals **(B,B1)**. The data are demonstrative of at least three independent experiments. **(A)** ***p < 0.001 vs. Sham; ^###^*p* < 0.001 vs. VaD; **(B)** ***p < 0.001 vs. Sham; ^###^*p* < 0.001 vs. VaD. Western blot analysis for iNOS and COX-2 showed a substantial increase of expression of these proteins in VaD mice compared to Sham; co-ultraPEALut treatment considerably reduced iNOS and COX-2 expression [respectively, **(C,C1,D,D1)**]. The data are illustrative of at least three independent experiments. **(C)** **p < 0.01 vs. Sham; ^###^*p* < 0.001 vs. VaD; **(D)** ***p < 0.001 vs. Sham; ^###^*p* < 0.001 vs. VaD. Data were determinated by one-way ANOVA followed by Bonferroni *post hoc* test.

NF-κB activation plays an important role in VaD. To determine the effect of co-ultraPEALut on the activation of NF-κB, we evaluated the cellular levels of IκB-α by Western blot analysis. In the Sham group, NF-κB is stabilized by IκB in the cytoplasm. After carotid arteries ligation, it triggers the degradation of IκB-α to free NF-κB for transport into the nucleus. In fact, our results showed that, following cerebral damage, the level of IκB-α protein was decreased in the cytoplasm (Figure 4A, see densitometry analysis A1 ***p < 0.001 vs. Sham; ^###^*p* < 0.001 vs. VaD), while high levels of NF-κB p65 were observed in the nucleus compared with Sham group (Figure 4B, see densitometry analysis B1 ***p < 0.001 vs. Sham; ^###^*p* < 0.001 vs. VaD). However, co-ultraPEALut treatment significantly prevented the degradation of IκB-α and the resulting NF-κB translocation in the nucleus, compared with the VaD group (Figures 4A,B, see respective densitometry analysis in A1 and B1).

### Effect of Co-ultraPEALut on iNOS and COX-2 Expression

To determine the role of ^⋅^NO produced after carotid arteries ligation, iNOS expression was evaluated using Western blot analysis. A significant increase in iNOS expression was observed in brain from mice subjected to carotid arteries ligation; while a significant decrease in iNOS expression was observed after co-ultraPEALut treatment (Figure 4C, see densitometry analysis C1 **p < 0.01 vs. Sham; ^###^*p* < 0.001 vs. VaD). Similarly, COX-2 expression was induced by carotid arteries ligation compared to the Sham group. Treatment with co-ultraPEALut significantly lowered COX-2 expression (Figure 4D, see densitometry analysis D1 ***p < 0.001 vs. Sham; ^###^*p* < 0.001 vs. VaD).

### Effects of Co-ultraPEALut on Bax and Bcl-2 Expression in Mice with VaD

**Figure 5:**
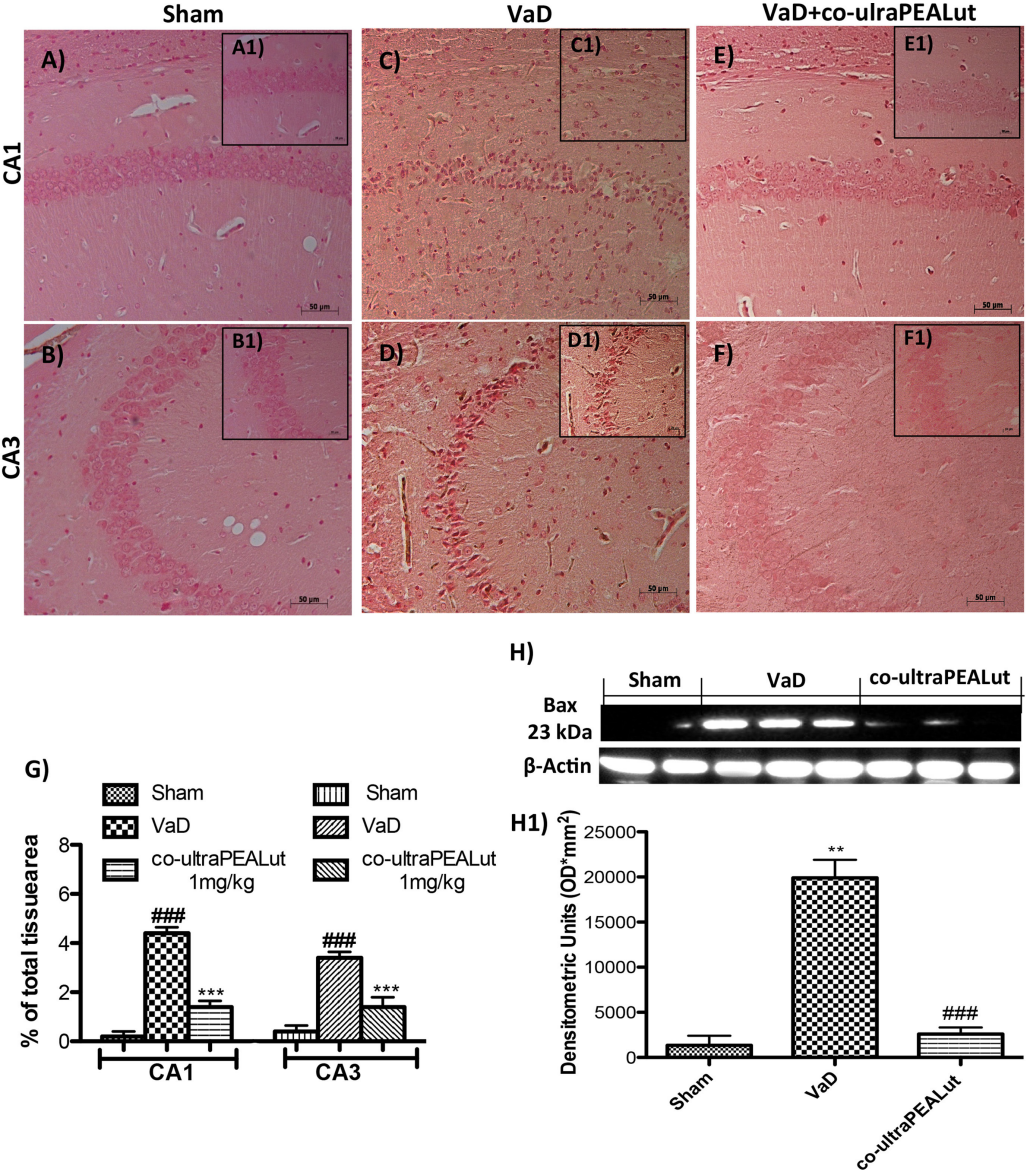
**Effects of co-ultraPEALut on proapoptotic protein in VaD mice**. Immunohistochemical analysis showed that Bax was significantly increased after carotid arteries ligation in hippocampal CA1 and CA3 regions [**(A,A1,B,B1)** vs. **(C,C1,D,D1,G)**]; instead, treatment with co-ultraPEALut restored Bax expression [**(C,C1,D,D1**) vs. (**E,E1,F,F1,G)**]. In addition, Western blot analysis demonstrated Bax expression to be significantly increased in VaD group, whereas treatment with co-ultraPEALut significantly reduced levels of Bad **(H,H1)**. The data are representative of at least three independent experiments. **(G)** ***p < 0.001 vs. Sham; ^###^*p* < 0.001 vs. VaD; **(H)** **p < 0.01 vs. Sham; ^###^*p* < 0.001 vs. VaD, as determined by One way ANOVA followed by Bonferroni *post hoc* test.

**Figure 6:**
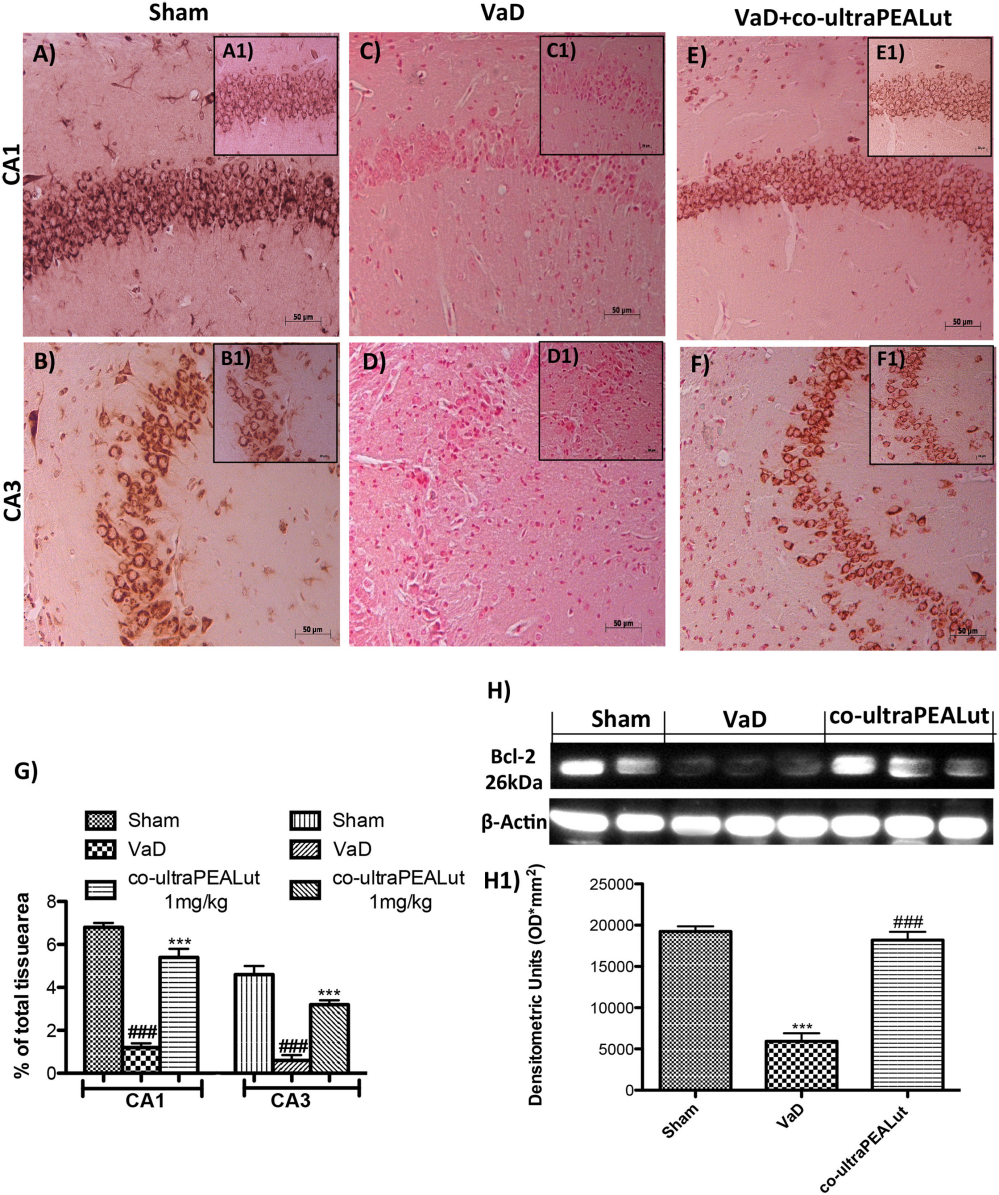
**Effects of co-ultraPEALut on antiapoptotic protein in VaD mice**. A significantly positive immunostaining for Bcl-2 was found in Sham group and in mice after co-ultraPEALut administration **(A,A1,B,B1,E,E1,F,F1,G)**, while VaD mice showed a decreased expression of Bcl-2 in hippocampal CA1 and CA3 regions **(C,C1,D,D1,G)**. Moreover, Western blot analysis demonstrated that Bcl-2 expression was reduced after carotid arteries occlusion; however, treatment with co-ultraPEALut restored basal levels **(H,H1)**. The data are illustrative of at least three independent experiments. **(G)** ***p < 0.001 vs. Sham; ^###^*p* < 0.001 vs. VaD; **(H)** ***p < 0.001 vs. Sham; ^###^*p* < 0.001 vs. VaD, as determined by one-way ANOVA followed by Bonferroni *post hoc* test.

To explore molecular mechanism underlying of the neuroprotective effect of co-ultraPEALut, we evaluated, in the mice hippocampal CA1 and CA3 regions, two proteins implicated in apoptotic death using immunohistochemical staining. Low Bax expression was found in the Sham group (Figures 5A,B, see magnification higher A1, B1, and relative densitometric analysis Figure 5G ***p < 0.001 vs. Sham; ^###^*p* < 0.001 vs. VaD). In the VaD group, we observed high Bax expression (Figures 5C,D, see magnification higher C1, D1, and relative densitometric analysis Figure 5G ***p < 0.001 vs. Sham; ^###^*p* < 0.001 vs. VaD); whereas after the co-ultraPEALut treatment Bax levels were significantly decreased (Figures 5E,F, see magnification higher E1, F1, and relative densitometric analysis Figure 5G ***p < 0.001 vs. Sham; ^###^*p* < 0.001 vs. VaD). To confirm immunohistochemical data, Western blot analysis was performed. The expression levels of Bax were significantly increased in the VaD group mice compared with those of the Sham group. By contrast, Bax expression was significantly reduced in mice treated with co-ultraPEALut (Figure 5H, see densitometric analysis H1 **p < 0.01 vs. Sham; ^###^*p* < 0.001 vs. VaD). On the contrary, using immunohistochemical staining we showed a low Bcl-2 expression in the Sham group (Figures 6A,B, see magnification higher A1, B1, and relative densitometric analysis Figure 6G ***p < 0.001 vs. Sham; ^###^*p* < 0.001 vs. VaD). In the VaD group, the immunostaining for Bcl-2 was reduced compared with the Sham group in the hippocampal CA1 and CA3 regions (Figures 6C,D, see magnification higher C1, D1, and relative densitometric analysis Figure 6G ***p < 0.001 vs. Sham; ^###^*p* < 0.001 vs. VaD). Instead, Bcl-2 levels were significantly increased in comparison with the VaD group after co-ultraPEALut treatment (Figures 6E,F, see magnification higher E1, F1, and relative densitometric analysis Figure 6G ***p < 0.001 vs. Sham; ^###^*p* < 0.001 vs. VaD). Using Western blot analysis, we showed that the expression levels of Bcl-2 were significantly reduced in the VaD group mice compared with Sham group; while levels of Bcl-2 were significantly increased in mice treated with co-ultraPEALut (Figure 6H, see densitometry analysis H1 ***p < 0.001 vs. Sham; ^###^*p* < 0.001 vs. VaD).

### Effects of Co-ultraPEALut on Nitrotyrosine and PAR Polymer

**Figure 7:**
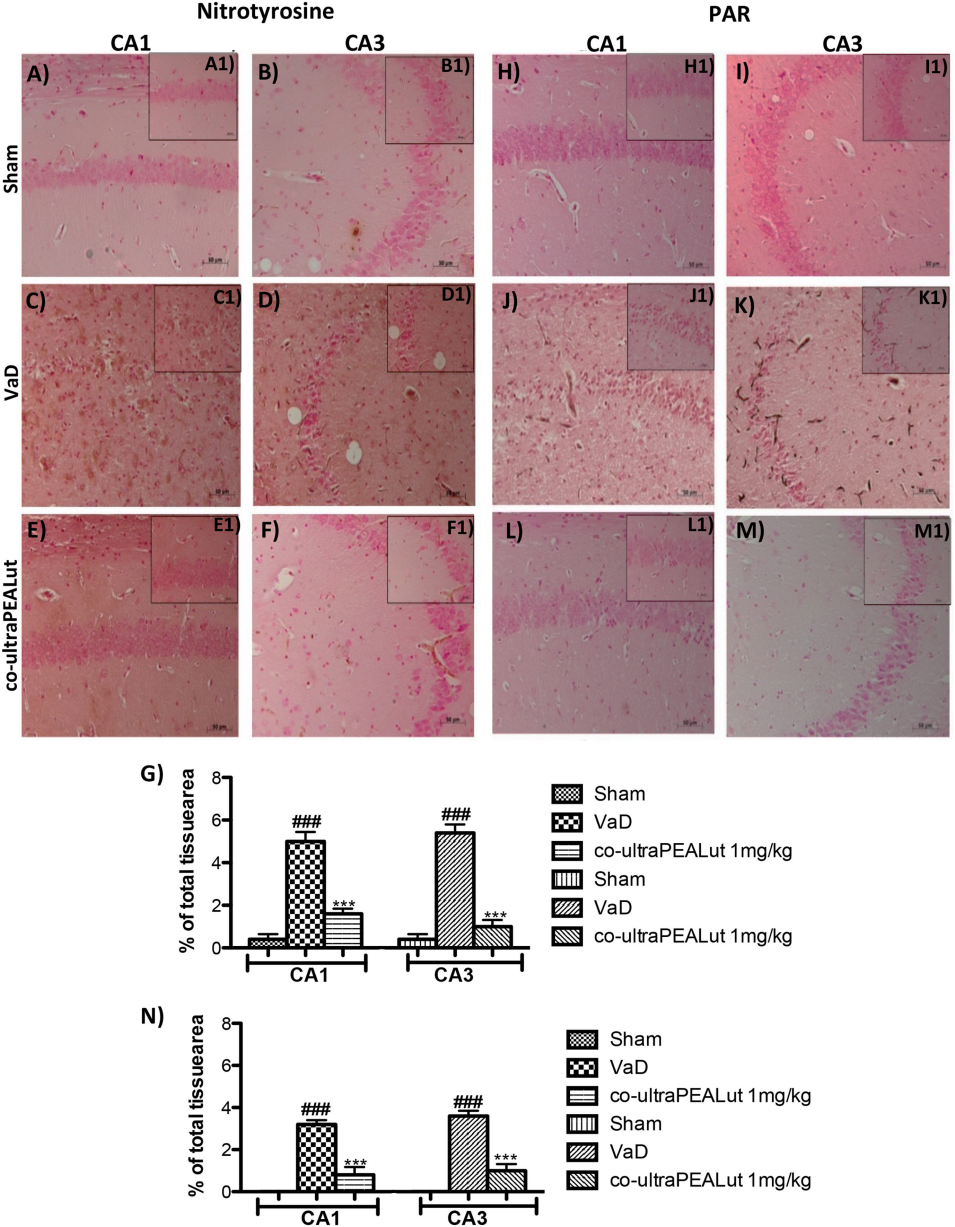
**Effects of co-ultraPEALut treatment on nitrotyrosine and PAR formation in VaD**. Brain sections from Sham mice did not stain for nitrotyrosine **(A,A1,B,B1,G)** and for PAR **(H,H1,I,I1,N)**, sections from VaD mice exhibited positive immunostaining for nitrotyrosine **(C,C1,D,D1,G)** and PAR formation **(J,J1,K,K1,N)**. Treatment with co-ultraPEALut reduced the degree of positive immunostaining for nitrotyrosine **(E,E1,F,F1,G)** and for PAR formation **(L,L1,M,M1,N)** in hippocampal CA1 and CA3 regions. **(G)** ***p < 0.001 vs. Sham; ^###^*p* < 0.001 vs. VaD; **(N)** ***p < 0.001 vs. Sham; ^###^*p* < 0.001 vs. VaD, as determined by one-way ANOVA followed by Bonferroni *post hoc* test.

Fifteen days after damage, nitrotyrosine, a specific marker of nitrosative stress, was investigated by immunohistochemical staining in the brain slices to determine the localization of “peroxynitrite formation” and/or other nitrogen derivatives formed following carotid arteries ligation. No positive staining for nitrotyrosine was detected in hippocampal CA1 and CA3 regions obtained from Sham mice (Figures 7A,B, see magnification higher A1, B1, and relative histological analysis Figure 7G ***p < 0.001 vs. Sham; ^###^*p* < 0.001 vs. VaD); whereas brain sections obtained from mice, that underwent occlusion of the carotid arteries, exhibited positive staining for nitrotyrosine (Figures 7C,D, see magnification higher C1, D1, and relative histological analysis Figure 7G ***p < 0.001 vs. Sham; ^###^*p* < 0.001 vs. VaD). Treatment with co-ultraPEALut significantly reduced the positive nitrotyrosine staining in hippocampal CA1 and CA3 regions (Figures 7E,F, see magnification higher E1, F1, and relative histological analysis Figure 7G ***p < 0.001 vs. Sham; ^###^*p* < 0.001 vs. VaD). In addition, a significant positive staining for the PAR was found primarily localized in nuclei of inflammatory cells in the brain tissues from the VaD group of mice (Figures 7J,K, see magnification higher J1, K1, and relative histological analysis Figure 7N ***p < 0.001 vs. Sham; ^###^*p* < 0.001 vs. VaD). Co-ultraPEALut reduced the degree of positive staining for PAR in the hippocampal CA1 and CA3 regions (Figures 7L,M, see magnification higher L1, M1, and relative histological analysis Figure 7N ***p < 0.001 vs. Sham; ^###^*p* < 0.001 vs. VaD). Note that there was no staining for PAR in brain tissues obtained from the Sham group of mice (Figures 7H,I, see magnification higher H1, I1, and relative histological analysis Figure 7N ***p < 0.001 vs. Sham; ^###^*p* < 0.001 vs. VaD).

### Effects of Co-ultraPEALut on Loss of Neuronal Cells after Carotid Arteries Occlusion

**Figure 8:**
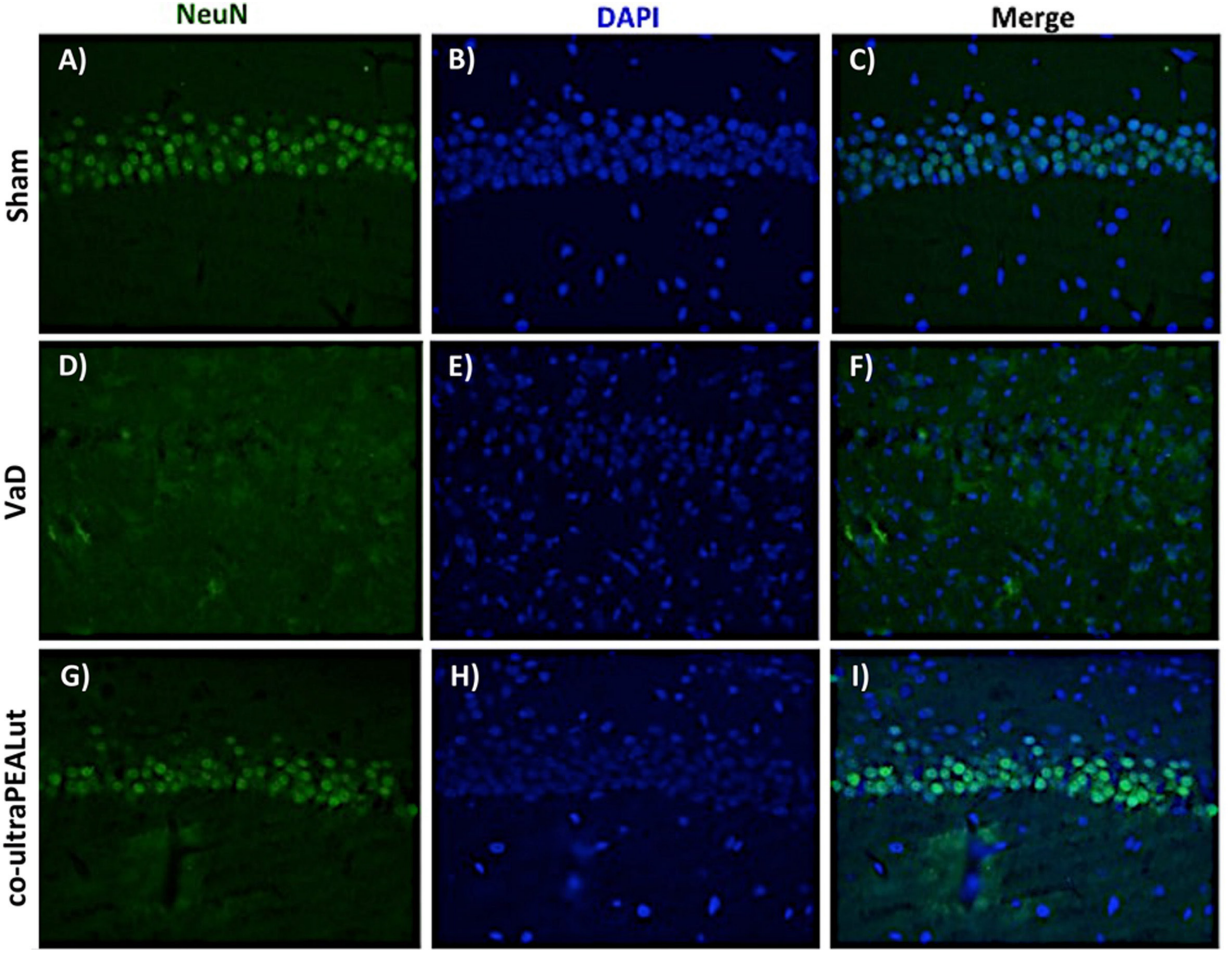
**Effects of co-ultraPEALut on neuronal cells after carotid arteries occlusion**. Results are shown for Sham group **(A–C)**, mice after carotid arteries ligation **(D–F)**, mice treated with co-ultraPEALut **(G–I)**. Representative images showing the intensity of fluorescent signals in regions labeled for NeuN and DAPI in the hippocampus from Sham animals **(A,B)**, animals submitted to carotid arteries occlusion **(D,E)**, and animals treated with co-ultraPEALut **(G,H)**. The co-localization between NeuN/DAPI is represented by the images **(C,F,I)**. Immunofluorescence staining showed that carotid arteries ligation decreased the NeuN immunocontent 15 days after surgery, while treatment with co-ultraPEALut restored the number of neuronal cells.

To investigate the effect of co-ultraPEALut on hippocampal damage accompanied by massive loss of neuronal cells in VaD mice, immunofluorescence staining was performed using anti-NeuN antibody. Our results showed an evident loss of NeuN-labeled viable neurons in the hippocampal region were observed in mice of VaD group (Figures 8D–F) in comparison with the Sham group (Figures 8A–C). However, this neuronal loss in VaD mice was reduced after co-ultraPEALut treatment (Figures 8G–I).

### Co-ultraPEALut Restores VaD-Induced Loss of BDNF and NT-3 Expression in Mice

**Figure 9:**
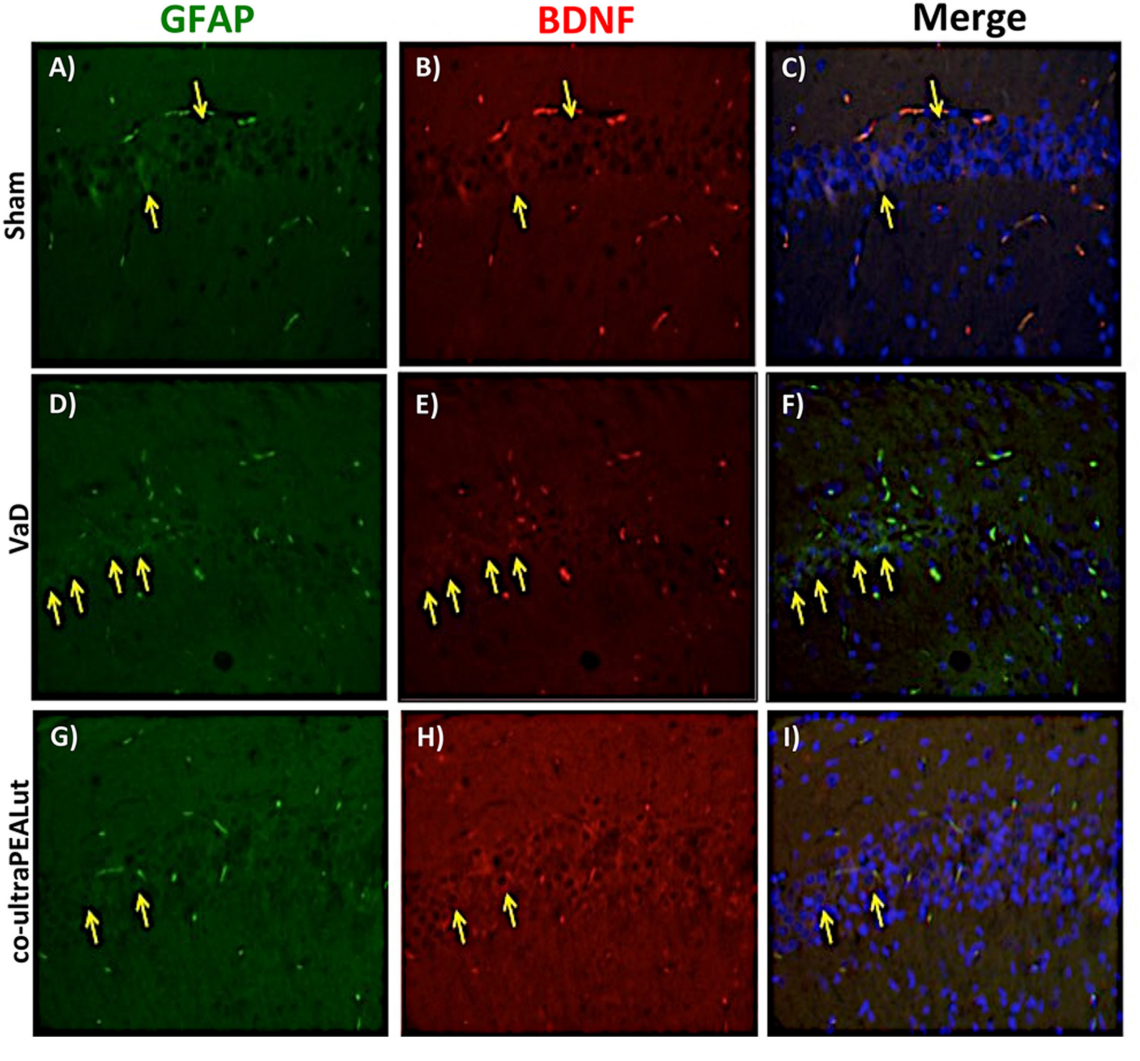
**Co-localization of GFAP/brain-derived neurotrophic factor (BDNF) after carotid arteries occlusion**. Results are shown for Sham group **(A–C)**, mice after carotid arteries occlusion **(D–F)**, mice treated with co-ultraPEALut **(G–I)**. Brain sections were double stained with antibodies against GFAP [**(A,D,G)**, green], and BDNF [**(B,E,H)**, red]. Hippocampal area revealed increased astrogliosis **(D)** in VaD group. GFAP immunoreactivity was reduced in co-ultraPEALut-treated mice **(G)**. Yellow spots indicate co-localizations and revealed a high co-localization between GFAP/BDNF double staining **(C,F,I)**. The pictures are demonstrative of at least three experiments executed on distinctive experimental days. Images are representative of all the animals in every group. All pictures were digitalized at a resolution of 8 bits into an array of 2,048 × 2,048 pixels.

**Figure 10:**
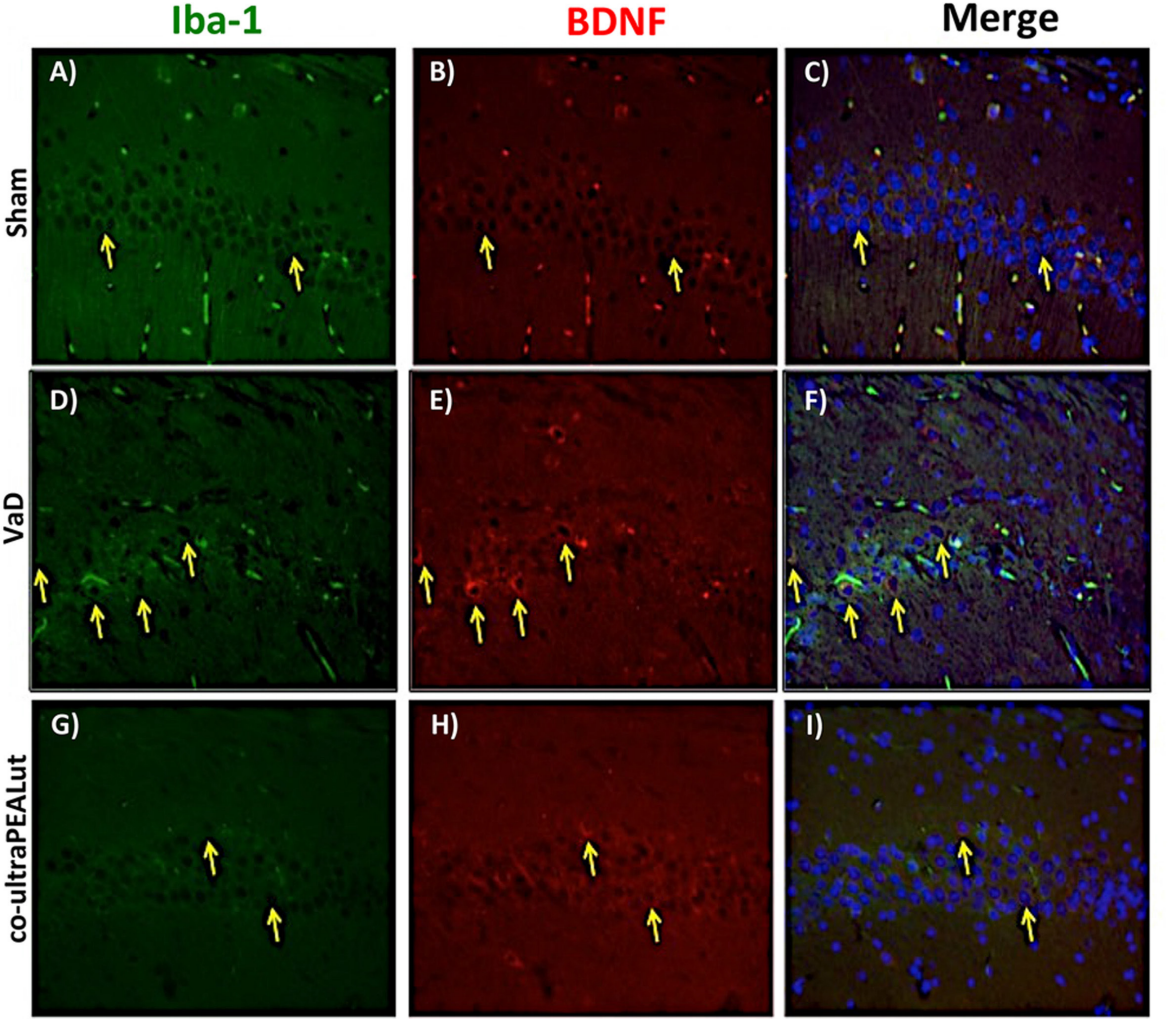
**Co-localization of Iba-1/brain-derived neurotrophic factor (BDNF) after carotid arteries occlusion**. Results are shown for Sham group **(A–C)**, mice after carotid arteries occlusion **(D–F)**, mice treated with co-ultraPEALut **(G–I)**. Brain sections were double stained with antibodies against Iba-1 [**(A,D,G)**, green] and BDNF [**(B,E,H)**, red]. Hippocampal area revealed increased microgliosis **(D)** in VaD group. Iba-1 immunoreactivity was reduced in co-ultraPEALut-treated mice **(G)**. Yellow spots indicate co-localizations and revealed a high co-localization between Iba-1/BDNF double staining **(C,F,I)**. The pictures are demonstrative of at least three experiments executed on distinctive experimental days. Images are representative of all the animals in every group. All images were digitalized at a resolution of 8 bits into an array of 2,048 × 2,048 pixels.

**Figure 11:**
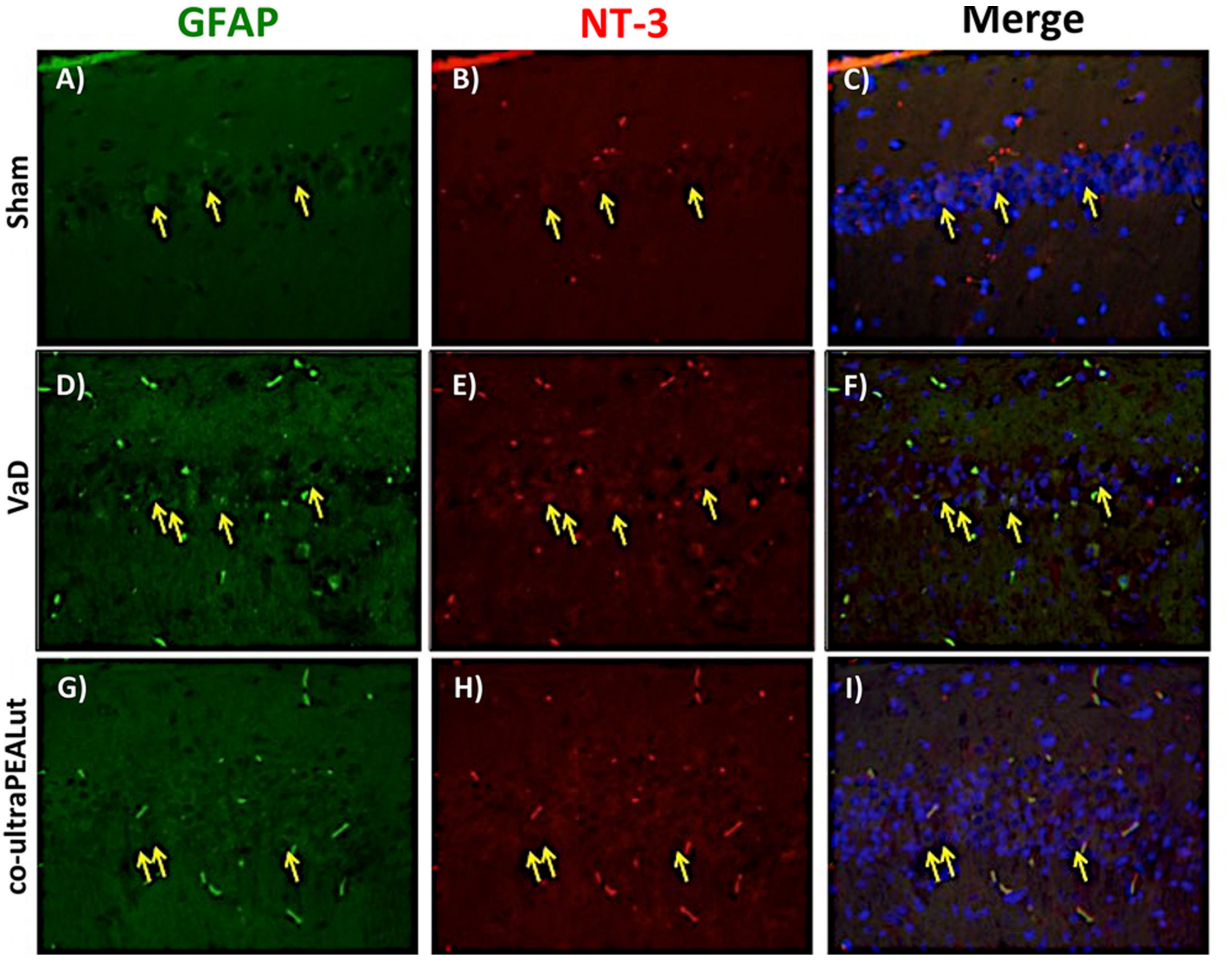
**Co-localization of GFAP/NT-3 after carotid arteries ligation**. Results are shown for Sham group **(A–C)**, mice after carotid arteries ligation **(D–F)**, mice treated with co-ultraPEALut **(G–I)**. Brain sections were double stained with antibodies against GFAP [**(A,D,G)**, green] and NT-3 [**(B,E,H)**, red]. Hippocampal sections revealed increased astrogliosis **(D)** in VaD group. GFAP immunoreactivity was reduced in co-ultraPEALut-treated mice **(G)**. Yellow spots indicate co-localizations and revealed a high co-localization between GFAP/NT-3 double staining **(C,F,I)**. The pictures are demonstrative of at least three experiments executed on distinctive experimental days. Images are representative of all the animals in every group. All images were digitalized at a resolution of 8 bits into an array of 2,048 × 2,048 pixels.

**Figure 12:**
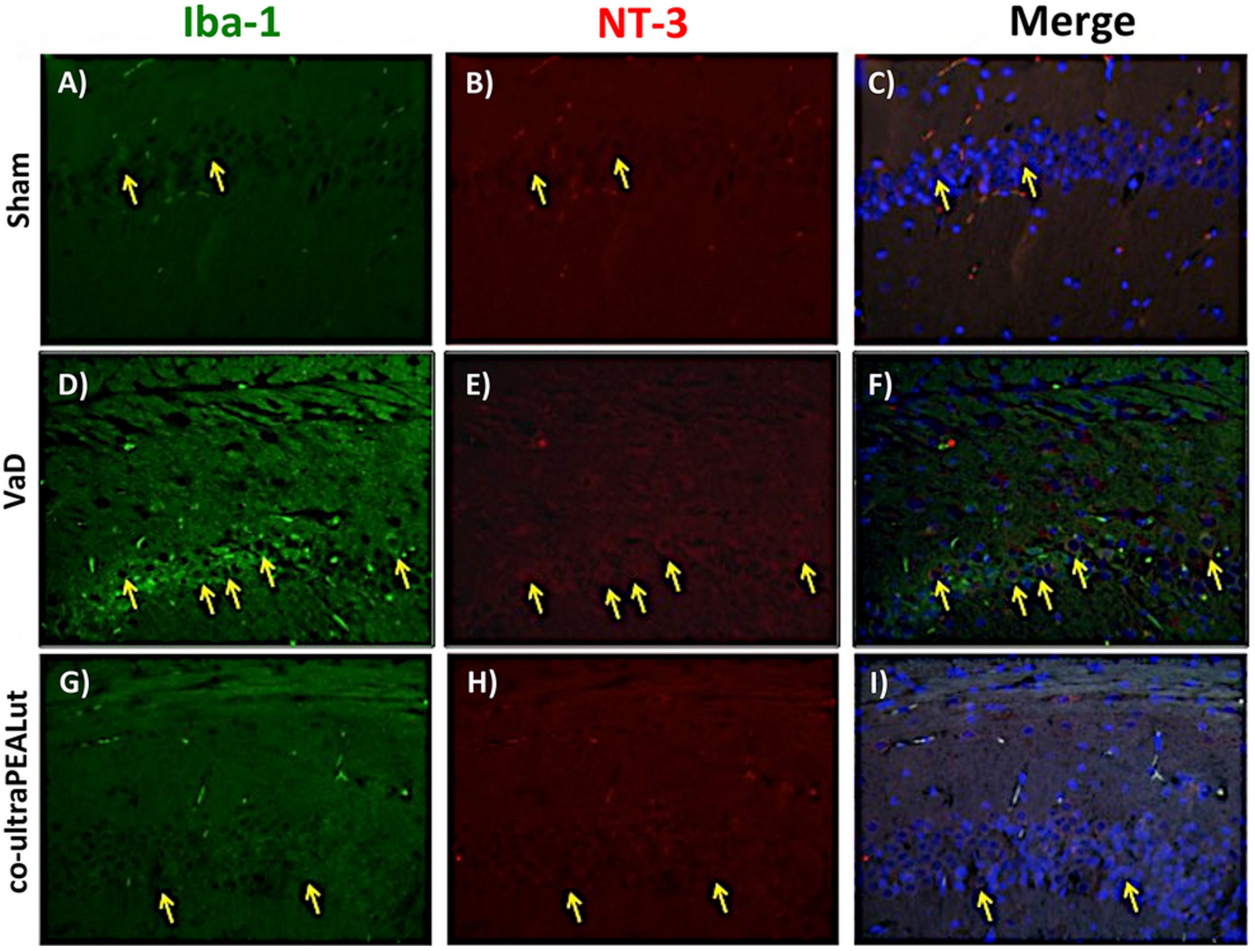
**Co-localization of Iba-1/NT-3 after carotid arteries ligation**. Results are shown for Sham group **(A–C)**, mice after carotid arteries ligation **(D–F)**, mice treated with co-ultraPEALut **(G–I)**. Brain sections were double stained with antibodies against Iba-1 [**(A,D,G)**, green] and NT-3 [**(B,E,H)**, red]. Hippocampal sections revealed increased microgliosis **(D)** in VaD group. Iba-1 immunoreactivity was reduced in co-ultraPEALut-treated mice **(G)**. Yellow spots indicate co-localizations and revealed a high co-localization between Iba-1/NT-3 double staining **(C,F,I)**. The pictures are demonstrative of at least three experiments executed on distinctive experimental days. Images are representative of all the animals in every group. All images were digitalized at a resolution of 8 bits into an array of 2,048 × 2,048 pixels.

The BDNF and NT-3 are essential to support the survival of existent neurons and to promote the growth and differentiation of novel neurons and synapses. To investigate whether co-ultraPEALut modulates the inflammatory process through regulation of the neurotrophic factors levels and to show their location in specific cell types, we performed immunofluorescence staining with Iba-1 and GFAP, microglial and astrocyte activation marker, respectively. Brain sections were double stained with antibodies against Iba-1 and GFAP (green) and BDNF and NT-3 (red). Immunofluorescence staining revealed that GFAP was significantly higher in mice of VaD group (Figures 9D and 11D) such as activation of the microglia (Figures 10D and 12D) than in the Sham group (Figures 9A, 10A, 11A, and 12A). GFAP and Iba-1 immunoreactivity were significantly reduced in mice treated with co-ultraPEALut (Figures 9G, 10G, 11G, and 12G). On the contrary, the neurotrophic factors were decreased in animals after carotid arteries ligation (Figures 9E, 10E, 11E, and 12E), respect the Sham group (Figures 9B, 10B, 11B, and 12B). Treatment with co-ultraPEALut significantly increased the release of BDNF and NT-3 (Figures 9H, 10H, 11H, and 12H). The yellow arrow indicates the co-localization between BDNF and GFAP (Figures 9C,F,I) and NT-3 and GFAP (Figures 11C,F,I) as well as between BDNF and Iba-1 (Figures 10C,F,I), and NT-3 and Iba-1 (Figures 12C,F,I). Reported images are representative of triplicate experiments. All pictures were digitalized at a resolution of 8 bits into an array of 2,048 × 2,048 pixels.

## Discussion

The growing importance of dementias phenomenon imposes an increasingly urgent need to gain more knowledge about this group of diseases, the techniques that allow earlier diagnosis and accurate, and primarily on preventive and therapeutic strategies acting on the mechanisms that underlie neuropathological cascade that leads to the death of neurons and the consequent progressive loss of cognitive abilities (43). For dementia refers to a clinical syndrome of acquired loss of cognitive and emotional skills, such as to interfere with the normal functions of everyday life and the quality of life itself (44). They distinguish primary or degenerative forms of dementia and secondary forms such as VaD among which the main one is that on ischemic vascular basis. VaD occurs when the blood supply that transporting oxygen to the brain is suddenly cut off. The absence of oxygen and nutrients generates a condition in which the reestablishment of circulation results in oxidative and stress inflammation with consequent damage to the involved tissues, instead of the normal functionality of the recovery. It is therefore the prospect of investigating new therapeutic strategies very interesting that modify the course of the dementia disease through neuroprotective mechanisms to reduce oxidative stress and chronic inflammation leading to neurodegeneration. Currently no medication has shown with confidence its effectiveness in treating VaD (45). PEA is a lipid mediator used in the clinic for its neuroprotective, antineuroinflammatory and analgesic properties (19, 46, 47). Investigations have been carried out to identify the molecular mechanism of action through which PEA exerts its pharmacological effects. This research has revealed that PEA can act via multiple mechanisms (47). The first mechanism of action for PEA was proposed by Rita Levi-Montalcini’s research group, who suggested that PEA acts via “Autacoid Local Injury Antagonism” to downregulate mast cell activation (48, 49). Later, the existence of a “direct receptor-mediated mechanism” was proposed, and several studies demonstrated that PEA can act via direct activation of at least two different receptors: the peroxisome proliferator-activated receptor α (PPAR-α) (50) and the orphan GPCR 55 (GPR55) (51). PPAR-α is expressed in many organs and tissues, such as the intestine, heart, liver, kidney, muscle, and adipose tissue, and also in several cells of the immune system (52). Although PPAR-α is the molecular target that directly mediates some of the neuroprotective, antineuroinflammatory, and analgesic effects of PEA (53), the existence of indirect mechanisms of action for this compound has also often been demonstrated. Therefore, a synergistic interaction can occur between the various mechanisms and explain why PEA has multiple effects and the ability to act on different cell types. Preclinical and human studies indicate that PEA, especially when co-micronized together with antioxidants, such as luteolin, and in micronized or ultramicronized forms, is a therapeutic tool with high potential for the effective treatment of different pathologies characterized by neurodegeneration, neuroinflammation, and pain (54). Previous studies have shown that PEA-luteolin combination can reduce inflammatory processes associated with spinal cord injury and TBI by restoring basal expressions of PPARs and by limiting NF-kB activation (31, 55). Moreover, co-ultraPEALut was demonstrated to exhibit neuroprotective effects in models of cerebral ischemia, Parkinson’s disease, and Alzheimer’s disease (29, 30, 37). However, its effects on VaD had not yet been evaluated. However, its effects on VaD had not yet been evaluated. Therefore, the purpose of this study was to explain the neuroprotective effects of co-ultraPEALut in a mouse model of VaD.

The experimental design was divided in two steps. First, we demonstrated by pharmacokinetic studies that the PEA, the major component of the compound co-ultraPEALut, is able to cross the blood-brain barrier. Second, we shown that the new compound co-ultraPEALut was able of improving the behavior and histopathological features in mice after VaD-induction. In addition, we observed that treatment with co-ultraPEALut had beneficial effects on inflammatory and apoptotic processes and on the response to oxidative and nitrosative stress. In detail in the first step, using LC-APCI-MS, we evaluated the PEA levels in the brain of the healthy rats. This analysis showed that after oral administration of 30 mg/kg of UM-PEA its levels were increased after 5 min and that its concentration peak was obtained after 15 min. Therefore, our results showed that PEA is able to penetrate into the brain.

Based on this evidence, in the second step, using H&E staining, we evaluated the effect of co-ultraPEALut on hippocampal CA1 and CA3 regions at 15 days after injury. Histological analysis showed that mice, after VaD, presented neuronal cell loss and abnormal architecture in hippocampal; while mice treated with co-ultraPEALut showed a significant decreased neuronal cell death and a marked reorganization in CA1 and CA3 regions. The memory is the cognitive domain most commonly damaged in dementia. In addition to memory, other higher faculties are affected in dementing process; they include language, visuospatial skills, calculation, and problem-solving skills. In many syndromes, dementigene also develop neuropsychiatric disturbances and alterations in social interactions, which result in depression, isolation social, hallucinations, delusions, agitation, and insomnia. For this reason in our study, we evaluated changes in cognitive function, reciprocal social interactions, and locomotor activity using specific behavioral tests. Using the Novel Object Recognition test, we demonstrated compromise of cognitive function because mice of VaD group showed significantly reduced preference for the new object; while treatment with co-ultraPEALut reduced the number of animals that had no interest for the novel object. Using social behavior test, we showed that time in the chamber with unfamiliar mouse was less in mice of VaD group, while in the group treated with co-ultraPEALut the number of animals who spent more time in the chamber with the unknown animal was greater. Finally, using an OF test we demonstrated an alteration of locomotor activity in mice of VaD group, because these animals spent less time in the center of arena, while this alteration was significantly reduced by treatment with co-ultraPEALut. The pathophysiological consequences of the discrepancy between demand and supply of energy substrates in the brain, for lack of arterial blood flow or reduced venous outflow, resulting in neuronal damage, derived from the impairment of cellular metabolism and the cascade of events resulting in the failure of cellular energy systems. These include: the release of excitotoxic mediators, inflammation and apoptosis (56). In this study, we examined the role of the proinflammatory NF-kB pathway in a mouse model of VaD. Using Western blot analysis, we demonstrated that, following carotid arteries ligation, the level of IκB-α protein was decreased in the cytoplasm, while high levels of NF-κB p65 were observed in the nucleus. However, co-ultraPEALut treatment significantly prevented the degradation of IκBα and the consequent migration of NF-κB in the nucleus. Other elements play a role in brain damage after carotid arteries ligation, such as activation of iNOS and COX-2 (57), increased expression of the proapoptotic molecule Bax and decreased expression of the antiapoptotic molecule Bcl-2 (58, 59); and PAR formation (60). In our study, co-ultraPEALut significantly reduced iNOS and COX-2 expression, normalized Bax/Bcl-2 expression levels and attenuated nitrotyrosine levels and PAR formation. Moreover, to study the changes of expression of NeuN in the hippocampus of mice with VaD we performed immunofluorescence staining. Compared with the Sham group, the expression of NeuN in CA1 and CA3 areas of hippocampus was significantly decreased in mice of VaD group 15 days after operation; while NeuN levels were increased with co-ultraPEALut treatment. Therefore, our results confirm the beneficial effect of the compound co-ultraPEALut on the loss of neuronal cells in the hippocampus. It is well know that neurotrophic factors such as BDNF and NT-3 regulate neuronal survival, axonal growth, synaptic plasticity, and neurotransmission that play an important role in reconstruction after injury in the adult CNS (61). Based on of these evidence, we evaluated the activity of co-ultraPEALut in limiting the loss of NT, so we have detected by immunofluorescence staining, that levels of BDNF and NT-3 were reduced in mice group VaD, while co-ultraPEALut treatment significantly increased the expression of these NT. In brain injuries, reactive astrocytes and microglia express low levels of BDNF and NT-3 (62, 63). In the present study, we observed the changes in GFAP and Iba-1 expression during brain damage, while co-ultraPEALut significantly prevented both astrogliosis and microgliosis. Furthermore, expression of BDNF, NT-3 was low in GFAP-positive astrocytes and in Iba-1-positive microglia. However, the expression of all two neurotrophic factors in GFAP and Iba-1 was noticeably induced after co-ultraPEALut treatment.

In conclusion, the present work represents the first description of co-ultraPEALut administration in an *in vivo* model of VaD. Taken together, our data clearly demonstrate that treatment with co-ultraPEALut reduced the inflammatory process, oxidative stress and the proapoptotic death that occur after carotid arteries occlusion. Thus, we propose that the compound co-ultraPEALut can be used as a treatment to control the cognitive impairment and the inflammatory and oxidative process associated with VaD.

## Author Contributions

Study concept and design: RS, SC, DI, and EE. Acquisition of data: RS, MC, RC, DI, and SP. Analysis and interpretation of the data: RS, DI, MC, RC, SC, EE, and SP. Drafting of the manuscript: RS, DI, and SC. Statistical analysis: RS, MC, RC, and SP. Critical revision of the manuscript for intellectual content: SC and EE. All authors read and approved the final manuscript.

## Conflict of Interest Statement

SC is co-inventor on patent WO2013121449 A8 (Epitech Group Srl), which treats with methods and compositions for the modulation of amidases capable of hydrolyzing *N*-acylethanolamines employable in the treatment of inflammatory diseases. This invention is wholly unrelated to the present study. Moreover, SC is also, with Epitech Group, a co-inventor on the following patent: EP 2 821 083; MI2014 A001495; 102015000067344, which are however unrelated to the study.
